# The updated national research agenda 2021–2026 for prehospital emergency medical services in the Netherlands: a Delphi study

**DOI:** 10.1186/s13049-021-00971-6

**Published:** 2021-11-20

**Authors:** Lilian C. M. Vloet, Gijs Hesselink, Sivera A. A. Berben, Margreet Hoogeveen, Paul J. T. Rood, Remco H. A. Ebben

**Affiliations:** 1 School of Health Studies, Research Department of Emergency and Critical Care, HAN University of Applied Sciences, PO Box 6960, 6503 GL Nijmegen, The Netherlands; 2 Radboud Institute for Health Sciences, IQ Healthcare, Radboud University Medical Center, Nijmegen, The Netherlands; 3 Dutch National Sector Organization for Ambulance Care (Ambulancezorg Nederland, AZN), PO BOX 4898000 AL, Zwolle, The Netherlands

**Keywords:** Ambulance, Emergency Medical Services, Evidence based practice, Research priorities, Delphi study

## Abstract

**Background:**

In 2015, a national research agenda was established for Dutch prehospital EMS to underpin the evidence base of care delivery and inform policymakers and funders. The continuously increasing demand for ambulance care and the reorientation towards the role of EMS in recent years may have changed research priorities. Therefore, this study aimed to update the Dutch national EMS research agenda.

**Methods:**

A three-round online Delphi survey was used to explore and discuss different viewpoints and to reach consensus on research priorities (i.e., themes and special interest groups, e.g. patient types who require specific research attention). A multidisciplinary expert panel (n = 62) was recruited in the field of prehospital EMS and delegates of relevant professional organizations and stakeholders participated. In round one, fifty-nine research themes and six special interest groups (derived from several resources) were rated on importance on a 5-point scale by the panel members. In round two, the panel selected their priority themes and special interest groups (yes/no), and those with a positive difference score were further assessed in round three. In this final round, appropriateness of the remaining themes and agreement within the panel was taken into account, following the RAND/UCLA appropriateness method, which resulted in the final list of research priorities.

**Results:**

The survey response per round varied between 94 and 100 percent. In round one, a reduction from 59 to 25 themes and the selection of three special interest groups was realized. Round two resulted in the prioritization of six themes and one special interest group ('Vulnerable elderly'). Round three showed an adequate level of agreement regarding all six themes: 'Registration and (digital) exchange of patient data in the chain of emergency care'; 'Mobile care consultation/Non conveyance'; 'Care coordination'; 'Cooperation with professional partners within the care domain'; 'Care differentiation' and 'Triage and urgency classification'.

**Conclusions:**

The updated Dutch national EMS research agenda builds further on the previous version and introduces new EMS research priorities that correspond with the future challenges prehospital EMS care is faced with. This agenda will guide researchers, policymakers and funding bodies in prioritizing future research projects.

**Supplementary Information:**

The online version contains supplementary material available at 10.1186/s13049-021-00971-6.

## Background

Emergency medical services (EMS) face a prehospital environment that is rapidly changing, characterized by an increasing number of ambulance deployments, due to a growing population with more complex healthcare problems and comorbidities, particularly for health problems that could be treated in the primary care [1–6]. As part of this increasing number of ambulance deployments there is also a significant number of patients that received ambulance care without conveyance [7–9]. The changing prehospital environment has led to an ongoing evolution of EMS worldwide. Although patient transport remains an important part of the provided service in all types of EMS systems, EMSs are evolving into an extended role of providers of prehospital advance care [10, 11] These changes have led to different types of ambulance care like medium care ambulances and psychiatric ambulances, the introduction of different types of prehospital care professionals like physician assistants and nurse practitioners, and the introduction of point of care testing, for example ultrasound and troponin tests [12–17].

Within the changing prehospital environment and the transformation of EMSs it is important that prehospital care remains effective, safe, and efficient. Scientific research is essential to evaluate the quality and safety of care, and to inform and direct professionals and policymakers in their efforts to improve patient outcomes [18, 19]. However, delivery of evidence-based prehospital care is increasingly complex due to a limited body of scientific knowledge available for the prehospital setting [20]. Moreover, this knowledge comes from research with serious limitations due to limited funding and research capacity, and methodological limitations such as difficulties to randomize and blind interventions and assess patient outcomes [21]. Furthermore, research in the prehospital setting is often challenging from an ethical perspective due to the context of urgency, time limitation and out-of-hospital locations of care delivery [22].

In order to support, further advance and focus research efforts, a national research agenda for prehospital EMS was developed [19]. This agenda was developed and based on the outcomes of a national Delphi study involving Dutch representatives from disciplines working in the prehospital EMS field and related stakeholders [19, 23]. From other healthcare domains we learn that a research agenda can be very helpful to target and stimulate research efforts [24–29] Despite the lack of evidence, a scan of EMS (scientific) research initiatives in recent years and the experiences shared by local EMS representatives indicate that a national research agenda has stimulated prehospital research in the Netherlands.

Yet, the changing prehospital environment, the evolving role of EMSs and current research deficits has increased a sense of urgency among EMS-professionals and policy-makers to investigate if previously set research priorities still correspond with current needs. Therefore, the aim of this study is to update the Dutch national EMS research agenda.

## Methods

### Design and setting

A three-round Delphi study was conducted in the Netherlands between September 2020 and January 2021 to obtain consensus on the opinions of a panel of experts through structured questionnaires [30], which enabled a structured information flow and adequate communication to all stakeholders. All participants remained anonymous during the study, which prevented that authority, status, personality, or reputation of group members influenced and potentially biased the process and outcomes. The study was executed according to the principles of the RAND/UCLA appropriateness method [30–32].

The study was commissioned by the Dutch National Sector Organization for Ambulance Care (AZN) and executed by an independent party, namely the research department of emergency and critical care of the HAN university of Applied Sciences, Nijmegen, the Netherlands to reduce the risk of bias in the inclusion of experts and the analysis of the data. In concordance with Dutch legislation, no approval of a medical ethical committee was needed.

### Delphi panel

AZN recruited experts having affinity with research, representing different areas of prehospital care and with different professional functions within ambulance care. Panel members were purposively sampled to compose a panel consisting of different types of EMS professionals within the prehospital EMS setting (i.e., internal stakeholders) across different geographical regions: medical managers (physicians) of ambulance care organizations, ambulance care professionals (nurses and bachelors of health), ambulance drivers, ambulance care dispatchers, physician assistants, nurse practitioners, EMS educators and researchers. In addition, mandated representatives of professional associations closely related to the field of EMS (i.e., external stakeholders), were recruited (e.g., emergency physicians, cardiologists, general practitioners and anesthesiologists) as well as delegates from network organizations, trade associations and external experts (researchers and policy staff). The Dutch Patients Federation was also invited to participate in the Delphi, but they decided not participate in the panel. This strategy resulted in a multidisciplinary panel of 62 experts (Additional file 1). In each Delphi round all panel members were contacted by the program manager research of AZN via an e-mail invitation. Non-responders were reminded once after two weeks.

### Data collection

The Delphi consisted of three consultation rounds using electronic surveys (Limesurvey version 3.26.2). In each round we provided the panel with feedback on the results of the previous consultation with the ultimate aim to reach consensus about a set of prehospital EMS research priorities which consisted of research themes (with an organizational and/or medical focus) as well as special interest groups (i.e., patient types who require specific research attention).

At the start, relevant research themes and special interest groups in prehospital EMS care were identified via several resources: 1) the previous version of the national research agenda; 2) five important documents regarding policy and vision on ambulance care at the national level published by EMS stakeholders (e.g., the Dutch Ministry of Health, branch associations and network organizations) between 2018 and 2020; 3) a scoping review by GH, RE, and LV of papers (i.e., reviews, original studies and opinion articles) on research in prehospital EMS care published between 2015 and 2020 in four high-impact journals (*Prehospital Emergency Care*, the *American Journal of Emergency Medicine*, *Scandinavian Journal of Trauma, Resuscitation and Emergency Medicine* and the *Journal of Emergency Medicine*); and 4) by consulting professionals in all 25 EMS districts about recently performed, current and planned studies via an online survey. We used multiple sources and involved different persons in the analysis of the data (GH, RE, MH, SB, LV) to identify a comprehensive set of relevant themes and special interest groups and to minimize risk of bias in selecting themes and special interest groups. Findings from all sources were synthesized into a framework of 59 different research themes across 12 categories (Table 1). Six special interest groups were identified (Table 1).

**Table 1:** Collected research themes and special interest groups

**A) Airway and breathing** Clearing the airway^A,B,C,D^ Ventilation (e.g. use of mask, balloon) ^A,B,C,D^ Auscultation^A,B,C,D^ Oxygen administration^A,B,C,D^ Medicinal intervention in COPD^A,B,C,D^ Treatment of hyperventilation^A,B,C,D^ **B) Circulation and Cardiology** Resuscitation^A,B,C,D^ Diagnosis and treatment of acute cardiac complaints^A,B,C,D^ Acute myocardial infarction (STEMI/ non STEMI) ^A,B,C,D^ Ultrasound^A,B,C,D^ Transfusion^A,B,C,D^ Shock therapy^A,B,C,D^ **C) Neurology and Anesthesiology** Pain (registration/treatment) ^A,B,C,D^ Recognition acute neurologic disorders^A,B,C,D^ CVA^A,B,C,D^ Intoxication (alcohol/drugs/medication) ^A,B,C,D^ Neurologic examination (Glasgow Coma Scale) ^A,B,C,D^ (Unintentional) cooling/hypothermia^A,B,C,D^ **D) Traumatology** Immobilisation^A,B,C,D^ Trauma care^A,B,C,D^ **E) Internal medicine** Acute abdominal complaints^A,B,C,D^ Screening and treatment of sepsis^A,B,C,D^ Diabetes^A,B,C,D^ Allergic reactions/ anaphylaxis^A,B,C,D^ **F) Psychiatry** Dealing with/treatment of confused behaviour^A,B,C,D^ **G) Gynaecology and Obstetrics** Child birth/partus^A,B,C,D^ Postpartum bleeding/ hemorrhage ^A,B,C,D^ **H) Organization of care** Mobile care consultation/ non-conveyance ^A,B,C,D^ Care stratification (Advanced life support, complexity, transports) ^A,B,C,D^ Differentiation in functions^A,B,C,D^ Cost-effectiveness (diagnostics, treatment and organization of care) ^A,B,C,D^ E-Health^A,B,C,D^ Triage and urgency clasification^A,B,C,D^ First responders (police, firemen, civilians) ^A,B,C,D^	'Rapid responders'^A,B,C,D^ Deployment of HEMS^A,B,C,D^ Deployment/ availability of ambulances^A,B,C,D^ Mass-casualty incident management^A,B,C,D^ **I) The chain of emergency care** Registration and exchange of patient data within the chain of emergency care^A,B,C,D^ Care coordination^A,B,C,D^ Interprofessional collaboration^A,B,C,D^ Collaboration with partners outside the healthcare domain (e.g. police, fire brigade) ^A,B,C,D^ Feedback/ evaluation of collaboration^A,B,C,D^ **J) Measuring quality of care** Patient safety^A,B,C,D^ Protocols (development, implementation and adherence) ^A,B,C,D^ Patient perspective and satisfaction^A,B,C,D^ 'Scoop and run'^A,B,C,D^ Registration and evaluation of time periods in ambulance deployment (response, treatment and transport time) ^A,B,C,D^ **K) Education/training of professionals** Professional behaviour Competences (knowledge, skills and attitude) ^A,B,C,D^ Forms of training (e.g. simulation, case-based learning) ^A,B,C,D^ E-Learning^A,B,C,D^ Testing and examining^A,B,C,D^ Clinical decision-making and diagnostic protocols) ^A,B,C,D^ **L) Human resources** Employee safety^A,B,C,D^ Vitality (physical and mental) and sustainable employability^A,B,C,D^ Ethics and spirituality^A,B,C,D^ Team climate/culture in EMS’s^A,B,C,D^ Recruitment, selection and retention of employees^A,B,C,D^ **Special Interest Groups** Vulnerable elderly Children Patients with a migration background Homeless Patients in a terminal/palliative phase Low Literacy

^A^Derived from the prior research agenda, ^B^Derived from scoping review, ^C^Derived from strategic documents, ^D^Derived from input of the expert panel. COPD = chronic obstructive pulmonary disease, STEMI = ST-Elevation Myocardial Infarction, CVA = Cerebrovasculair Accident, HEMS = Helicopter Emergency Medical Service

During round one, panel members were requested to rate the importance of all 59 themes and 6 special interest groups on a 5-point scale ranging from 1 (not important) to 5 (very important). We started with this pre-selected set of themes and groups, instead of an inventory round using unrestricted input from panel members like in the classical Delphi, because we reached data saturation during the identification process of the themes.

Additionally, panel members were invited (and not obliged) to formulate a maximum of three themes and special interest groups that did not appear in the list and to assess them in the same way to ensure that important themes and groups were not left out.

In round two, the 25 highest ranked themes and the 3 highest ranked special interest groups from round 1 were re-submitted to the panel. Members were asked again to prioritize themes and special interest groups by answering yes/no per theme and group. Additionally, participants could re-select one theme and one special interest group that was discarded or additionally identified after round 1.

In the final round three, the panel was asked to rate the importance of the remaining themes and groups on a 9-point Likert scale from 1 (not important) to 9 (very important). This enabled determination of the level of importance and agreement among the panel, in concordance to the RAND/UCLA appropriateness method [33]. Furthermore, panel members were asked to divide an amount of €100 over the research themes, which provided additional prioritizing based on a limited budget (scarcity), if themes would gain equal priority.

### Analysis

In round 1, all 59 themes and six special interest groups were ranked on importance, based on a calculated priority score i.e. the number of positive ratings (score 4 and 5) minus the number of negative ratings (score 1 and 2). In this way, both positive and negative opinions are taken into account [21, 22]. The additional self-formulated themes and special interest groups were analyzed and grouped by the researchers by looking at the formulation merging those with the same meaning. The themes and groups were subsequently ranked from most important to least important. To reduce the number of research themes, the first 25 themes that the panel scored as most important progressed to Delphi round 2. The threshold of 25 themes was predetermined in consultation with the AZN scientific committee. The three most important special interest groups were selected for assessment in the second round.

In round 2, the remaining 25 themes and three special interest groups were ranked based on a difference score calculated by: the frequencies of 'yes' per theme and special interest group (should be placed on the updated research agenda) minus the 'no' frequencies (should not be placed on the updated research agenda).

Themes and special interest groups were ranked from most important to least important. Themes and special interest groups with a positive difference score (more panelists found that a theme or special interest group should be on the agenda than not) progressed to Delphi round 3.

In round three, the RAND-UCLA/RAM method was used to determine the themes of the updated research agenda, following the classification of appropriateness and agreement [23]. Median scores were calculated for each theme. Scores between 7 and 9 were defined as appropriate, 4 to 6 as somewhat appropriate, and 1 to 3 as not appropriate. To determine agreement among participants of the Delphi panel on these themes, the disagreement index was calculated for each topic, and a disagreement index below 1 was regarded as adequate [23]. Finally, all themes were categorized based on the median score ranking the importance of the theme combined with the disagreement index. We defined three categories: (1) the theme is appropriate (median of 7–9) and there is consensus within the panel; (2) the theme is possibly appropriate (median 7–9), however without consensus or the theme is somewhat appropriate (median 4–6) with or without consensus in the panel; and, (3) the theme is not appropriate (median 1–3) (with or without consensus in the panel) [20]. All topics within the first category progressed to the final list of prehospital EMS research priorities. Data was processed using Microsoft Excel 2016 (Microsoft, USA).

## Results

The Delphi rounds yielded a response rate of 100% (n = 62) in round 1 and 2, and 94% (n = 58) in round 3. Reasons for non-response were not inquired.

**Fig. 1:**
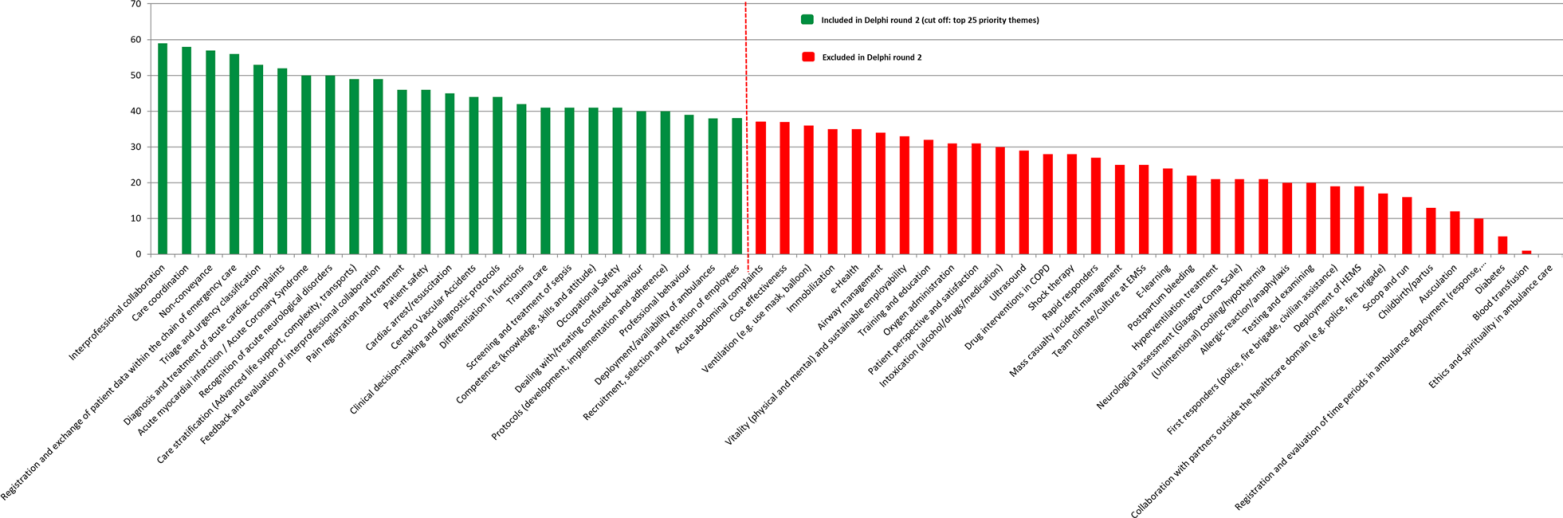
Research themes priorities (Delphi round 1)based on difference scores (very) important – (very) unimportant (n = 62)

**Fig. 2:**
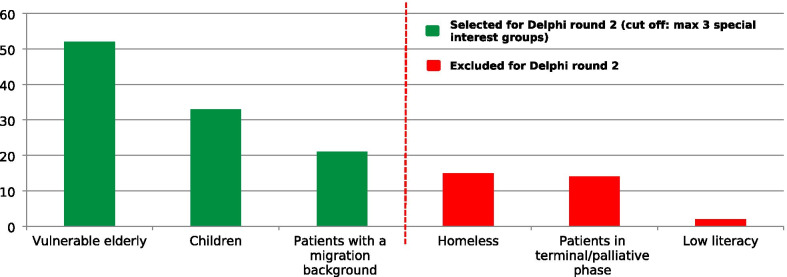
Priorities of special interest groups Delphi-round 1, based on difference scores (very) important – (very) unimportant (n = 62)

Figures 1 and 2 present the research themes and special interest groups ranked from most important to least important for round 1. Forty-two narratives were provided by panelists to describe additional themes. After merging these narratives the following additional themes were identified: 1) Syncope, 2) Medication safety, 3) Joint decision-making and 4) Quality of instructions by the EMS dispatcher. After Delphi round 1 starting with 59 themes, reduced to 25, minor adjustments were made in the wording of eight themes to improve clarity and the meaning of these themes after comments made by several panelists. Panelists assessed ‘Vulnerable elderly’, ‘Children’, and ‘Patients with a migration background’ as most important special interest groups. Thirty-two narratives were provided by panelists to describe additional special interest groups. After merging these narratives the following additional special interest groups were identified: (1) Persons with psychiatric problems, (2) Persons with (possibly) highly contagious diseases, and (3) Persons with multi morbidity.

**Fig. 3:**
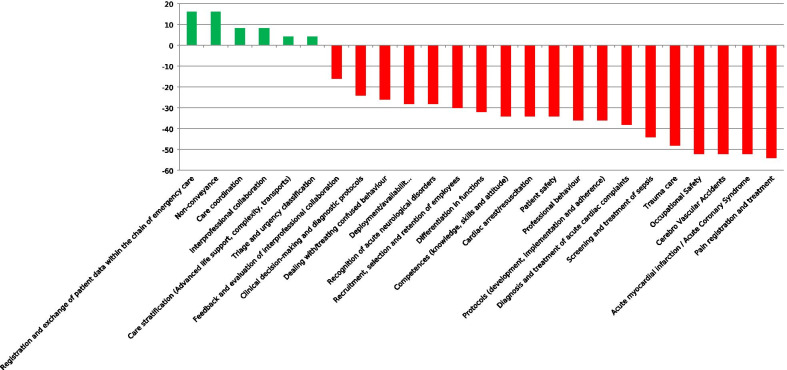
Research themes priorities (Delphi round 2), based on difference scores (very) important (very) unimportant (n = 62)

**Fig. 4:**
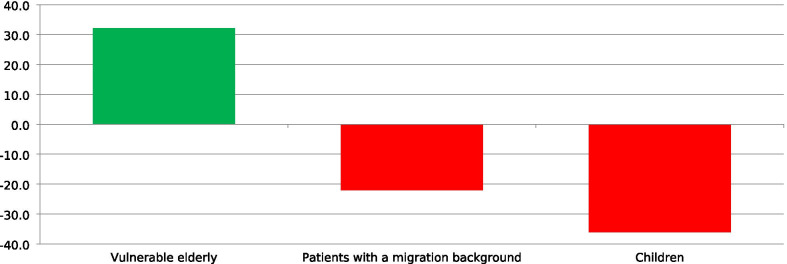
Priorities of special interest groups Delphi-round 2, based on difference scores (very) important – (very) unimportant (n = 62)

In round 2, the number of research themes was reduced from 25 to six (Fig. 3). Forty-four of 62 panelists (71%) re-added a research theme that was discarded after round 1. Twenty-five of 62 panelists (40%) re-added a research theme that was additionally identified after round 1. None of the re-added themes obtained a positive difference score which resulted in six themes that were assessed in round 3. The number of special interest groups was reduced from three to one (Fig. 4). Solely the special interest group ‘Vulnerable elderly’ achieved a positive difference score. Twenty-one of 62 panelists (34%) re-added a special interest group that was discarded after round 1. Twenty-four of 62 panelists (39%) re-added a group that was additionally identified after round 1. None of the re-added groups obtained a positive difference score. Therefore, the special interest groups were not further prioritized in Delphi round 3.

**Fig. 5:**
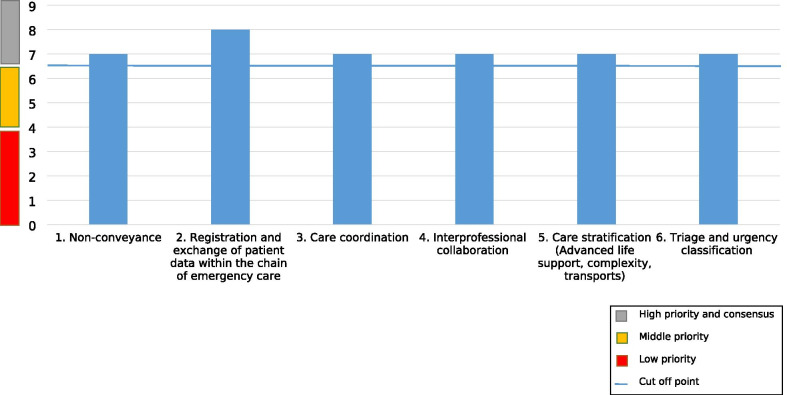
Scores (median, range 1–9) of the research themes in Delphi round 3 (n = 58)

The results of round 3 are presented in Fig. 5. The disagreement index, the measure for consensus, was below 1 for all six research themes indicating an adequate level of agreement within all themes. Moreover, all six themes were rated as appropriate for the updated research agenda because these themes were rated a median of 7 or higher. Median scores were all between 7 and 9, indicating appropriateness. The theme 'Registration and digital exchange of patient data in the acute care chain' received the highest rank. Consequently, six themes were added to the updated national prehospital EMS research agenda: (1) 'Registration and digital exchange of patient data in the acute care chain'; (2) 'Mobile care consultation/Non conveyance; (3) 'Care coordination'; (4) 'Cooperation with partners within the care domain'; (5) 'Care differentiation'; and (6) 'Triage and urgency classification'.

## Discussion

This study updated the national research agenda for prehospital EMS in the Netherlands, which was first developed in 2015, by determining new research priorities. In a three-round Delphi study the panel gained adequate levels of agreement and prioritized six research themes and one special interest group.

Our study shows that several research themes (i.e., non-conveyance to the hospital, triage, and the registration and exchange of patient data in the acute care chain) still remain a priority as assessed by Dutch EMS professionals and related stakeholders, also six years after the first research agenda was developed [19]. On the contrary, we also observed shifts in research priorities. Firstly, a shift from medically oriented themes (e.g., assessment of acute neurologic signs and symptoms) towards themes related to the organization of acute care (e.g., care coordination and care differentiation). Second, a shift from research themes within EMS setting towards themes covering the chain of emergency care and collaboration between acute care services. These observed shifts are in line with current developments in the field of EMS care showing concentration and specialization of hospital care versus a shift of medical care provision outside the hospital. Furthermore, a change in the distribution of chronic, acute and elective care and care outside the hospital, leading to increasing complex care needs. This underpins the need for more coordination of (prehospital) emergency care and mobile care provision, collaboration in the chain of emergency care, and differentiation of care tasks and professionals.

The priority on vulnerable older persons as a special interest group in EMS research shows the actual need for evidence-based assessment, interventions, tools and geriatric skills experienced by EMS professionals to provide adequate care to this growing population with often complex needs. Geriatric emergency care is internationally regarded as an important research topic and involves different aspects that are potentially relevant for EMS professionals such as training geriatric skills and use of screening instruments [34].

The updated Dutch national prehospital EMS research agenda contains broadly defined research themes and many themes are intertwined. For the next step the prioritized themes need to be translated into actual research questions. Dissemination and implementation of the updated agenda requires careful attention and could be facilitated by the Dutch EMS field embracing and adhering to the implementation plan that is developed after the first research agenda in 2015. The updated research agenda shows the need for investing in close collaboration between EMS and other stakeholders in the chain of emergency care to successfully implement joint research initiatives.

To our knowledge, this is the first updated national research agenda in the field of EMS care. Our findings contribute to structural monitoring and frequent evaluation of research priorities in EMS care, which is important as the context of emergency care remains rapidly changing, and may guide other researchers in their efforts to update similar agenda’s in other countries [34, 35].

### Strengths and limitations

A strength of this study is the high response of the multidisciplinary expert panel in all rounds of the study. This indicates the continuing commitment and support for the national research agenda. Also, limitations of this study need to be addressed. First, we could not include the patient representative in the expert panel while the patient perspective is increasingly considered important in the design, execution and evaluation of scientific research. Although several attempts were made to include a patient representative in the panel, none of the invitations of the national patients federation were accepted. This reflects the difficulties for patient participation, especially in the EMS setting [36]. Second, the identified research priorities were gathered from a national expert panel whose experiences are bound to the country-specific context and care system. Findings may correspond with international research priorities, but are not necessarily generalizable to other countries. Third, the panel partly consisted of experts who combine clinical EMS work with a representative function in a national committee or association. This might have influenced the prioritization of themes and groups by the panel and might explain the shift from medically oriented themes in the previous agenda to themes related to the organization of acute care in the updated version. However, the observed shift is most likely caused by an an actual different prioritizement of themes as previous Delphi panel also partly consisted of experts with a combined function.

## Conclusions

Six research themes have been selected for the updated National Research Agenda Ambulance Care 2021–2026: 'Registration and (digital) exchange of patient data in the chain of emergency care', 'Mobile care consultation/Non conveyance', 'Care coordination', 'Cooperation with partners within the care domain', 'Care differentiation' and 'Triage and urgency classification'. In addition, the group of 'Vulnerable elderly' has been defined as a 'special interest group'. Based on this updated National Research Agenda Ambulance Care 2021–2026, priorities have been set with regard to scientific research within ambulance care in order to further professionalize it, stimulate knowledge creation and dissemination in the sector and strengthen the evidence base of the provision of care. These themes may serve as guidance for researchers, policymakers and funding bodies in prioritizing future research projects.

## Supplementary Information

**Additional file 1**: Delphi panel experts (n = 62).

## Data Availability

The datasets used and/or analyzed during the current study are available from the corresponding author on reasonable request.

## Abbreviations

AZN: Dutch National Sector Organization for Ambulance Care
EMS: Emergency Medical Service(s)
