# Supplementary material for: The updated national research agenda 2021–2026 for prehospital emergency medical services in the Netherlands: a Delphi study
Source: Scand J Trauma Resusc Emerg Med. 2021 Nov 20;29:162. doi: 10.1186/s13049-021-00971-6 (PMC8605575; doi:10.1186/s13049-021-00971-6)
Supplement: Supplementary file 1 — Additional file 1: Delphi panel experts (n = 62). [file 13049_2021_971_MOESM1_ESM.docx]

**Supplemental file 1 – Delphi panel experts (n=62)**

| Internal stakeholders | |
| --- | --- |
| Dutch National Sector Organization for Ambulance Care, department of research policy and staff | 4 |
| Dutch National Sector Organization for Ambulance Care, advisory board for care policy | 7 |
| Dutch Academy for Ambulance care | 2 |
| Dutch National Sector Organization for Ambulance Care, representative board committee chain quality | 6 |
| Dutch National Sector Organization for Ambulance Care, representative protocols committee | 4 |
| Dutch National Sector Organization for Ambulance Care, representative Science Committee | 5 |
| Dutch Association of Physician Assistants (NAPA) (representative) | 1 |
| Dutch Association of Bachelor Medical Caregivers (NVBMH) (representative) | 2 |
| Dutch Association of Medical EMS Managers (NVMMA) (representative) | 5 |
| Dutch Association of Nurses and Caregivers Netherlands (V&VN), department of EMS nurses | 4 |
| Dutch Association of Nurses and Caregivers Netherlands (V&VN), department of dispatchers | 2 |
| Dutch Association of Nurses and Caregivers Netherlands (V&VN), department of EMS drivers | 3 |
| Dutch Association of Nurses and Caregivers Netherlands (V&VN), department of nurse specialists | 5 |
| Dutch Association of Nurses and Caregivers Netherlands (V&VN), department of care ambulances | 2 |
|  | |
| External stakeholders | |
| Dutch Association of Emergency Physicians (NVSHA) (representative) | 1 |
| Dutch Association of Anesthesiology (NVA) (representative) | 1 |
| Dutch Association of Cardiology (NVVC) (representative) | 1 |
| Dutch Association of Intensive Care (NVIC) (representative) | 1 |
| Dutch Association of clinical geriatrics (NVKG) (representative) | 1 |
| Dutch Association of Obstetics and Gynaecology (NVOG) | 1 |
| Dutch Association of Trauma Surgery (NVT) | 1 |
| Zorgverzekeraars Nederland (ZN) as the umbrella organization health insurers in The Netherlands | 1 |
| Dutch National Network for Acute Care (LNAZ) | 1 |
| Trade association for elderly care (ACTIZ) (representative) | 1 |
